# Predicting Drug–Side Effect Relationships From Parametric Knowledge Embedded in Biomedical BERT Models: Methodological Study With a Natural Language Processing Approach

**DOI:** 10.2196/67513

**Published:** 2025-07-10

**Authors:** Woohyuk Jeon, Minjae Park, Doyeon An, Wonshik Nam, Ju-Young Shin, Seunghee Lee, Suehyun Lee

**Affiliations:** 1 Department of Computer Engineering, College of IT Convergence, Gachon University, AI·Engineering Building, 317A, 1342 Seongnam-daero, Sujeong-gu, Seongnam-si, Gyeonggi-do, Seongnam, 13120, Republic of Korea, 82 010-9012-9364, 82 031-750-5333; 2 School of Pharmacy, Sungkyunkwan University, Suwon, Republic of Korea; 3 Konyang Medical Data Research Group, Konyang University Hospital, Daejeon, Republic of Korea

**Keywords:** adverse drug reaction, ADR prediction, NLP, BERT, word embedding, drug-side effect relationship, Bidirectional Encoder Representations from Transformers, natural language processing

## Abstract

**Background:**

Adverse drug reactions (ADRs) pose serious risks to patient health, and effectively predicting and managing them is an important public health challenge. Given the complexity and specificity of biomedical text data, the traditional context-independent word embedding model, Word2Vec, has limitations in fully reflecting the domain specificity of such data. Although Bidirectional Encoder Representations from Transformers (BERT)–based models pretrained on biomedical corpora have demonstrated high performance in ADR-related studies, research using these models to predict previously unknown drug–side effect relationships remains insufficient.

**Objective:**

This study proposes a method for predicting drug–side effect relationships by leveraging the parametric knowledge embedded in biomedical BERT models. Through this approach, we predict promising candidates for potential drug–side effect relationships with unknown causal mechanisms by leveraging parametric knowledge from biomedical BERT models and embedding vector similarities of known relationships.

**Methods:**

We used 158,096 pairs of drug–side effect relationships from the side effect resource (SIDER) database to generate an adjacency matrix and calculate the cosine similarity between word embedding vectors of drugs and side effects. Relation scores were calculated for 8,235,435 drug–side effect pairs using this similarity. To evaluate the prediction accuracy of drug-side effect relationships, the area under the curve (AUC) value was measured using the calculated relation score and 158,096 known drug–side effect relationships from SIDER.

**Results:**

The clagator/biobert_v1.1 model achieved an AUC of 0.915 at an optimal threshold of 0.289, outperforming the existing Word2Vec model with an AUC of 0.848. The BERT-based models pretrained on the biomedical corpus outperformed the vanilla BERT model with an AUC of 0.857. External validation with the FDA (Food and Drug Administration) Adverse Event Reporting System data, using Fisher exact test based on 8,235,435 predicted drug–side effect pairs and 901,361 known relationships, confirmed high statistical significance (*P*<.001) with an odds ratio of 4.822. In addition, a literature review of predicted drug–side effect relationships not confirmed in the SIDER database revealed that these relationships have been reported in recent studies published after 2016.

**Conclusions:**

This study introduces a method for extracting drug–side effect relationships embedded in parameters of language models pretrained on biomedical corpora and using this information to predict previously unknown drug–side effect relationships. We found that BERT-based models pretrained with biomedical corpora consider contextual information and achieve better performance in drug–side effect relationship prediction. External validation using the FDA Adverse Event Reporting System dataset and the literature review of certain cases confirmed high statistical significance, demonstrating practical applicability. These results highlight the utility of natural language processing–based approaches for predicting and managing ADR.

## Introduction

An adverse drug reaction (ADR) is a harmful, unintended reaction that occurs despite the proper use of medication [1]. In addition to causing serious health problems, ADRs are known to be one of the leading causes of prolonged patient hospitalization and increased health care spending [2]. Approximately 2 million cases of serious ADRs are reported annually in the United States, resulting in 100,000 deaths [3]. Therefore, early prediction and prevention of ADRs during drug development is a critical challenge for patient safety and public health.

Traditionally, ADR prediction has been based on approaches that analyze the chemical structure, mechanism of action, and pharmacokinetic properties of drugs [4]. Subsequently, ADR prediction methodologies using machine learning techniques have been developed [5-7], and with advances in natural language processing (NLP) techniques, attempts have been made to automatically extract and predict drug-side effect relationships from vast amounts of biomedical literature data [8-10]. The prediction of ADRs using these techniques is accelerating, especially with the advent of word embedding methods such as Word2Vec [11], which can effectively vectorize semantic information embedded in textual data [12,13].

However, biomedical text data are characterized by a much more specialized and complex set of terms and concepts compared with the general literature, and the interactions between them are also highly diverse and dynamic [14]. In fact, it has been pointed out that traditional word embedding models such as Word2Vec, which do not consider contextual information, do not sufficiently represent the relationships between complex biomedical concepts [15,16]. Therefore, models that do not adequately reflect domain specificity are limited in their ability to accurately capture drug–side effect relationships.

One solution to this problem is to use language models based on Bidirectional Encoder Representations from Transformers (BERT) [17] to perform word embedding. BERT is a language model based on the transformer [18] architecture, which has recently gained attention; unlike traditional one-way language models, it has richer language expressiveness by learning context in both directions. In addition, because we trained on large corpora, domain-specific pretrained models using large biomedical corpora can fully reflect the domain specificity of the biomedical text data [19-22]. Recently, several BERT-based models have been proposed that use large biomedical corpora such as PubMed and PMC for domain-specific pretraining. Examples include BioBERT [19], BioMedBERT [20], and PharmBERT [23], which have demonstrated high performances in various bio-NLP tasks.

BERT models and BERT-based models pretrained on biomedical corpora have demonstrated high performance in ADR-related studies [22,24-27]. However, there is a lack of research leveraging these models to predict previously unknown drug–side effect relationships. Therefore, this study aims to use a biomedical domain-specific BERT language model based on the ADR prediction relation score methodology proposed by Seungsoo et al [12]. Specifically, we calculate the similarity between embedding vectors of known drug–side effect relationships and derive promising candidates for potential relationships. In other words, our objective is to efficiently identify drug–side effect relationships whose causal associations have not yet been clearly established, by computing relation scores from biomedical language model embeddings grounded in known relationships. Furthermore, we examine whether replacing the Word2Vec model with a BERT-based model leads to an actual improvement in ADR prediction accuracy, thus validating the performance advantages of context-dependent language models.

## Methods

### System Overview

**Figure 1.**
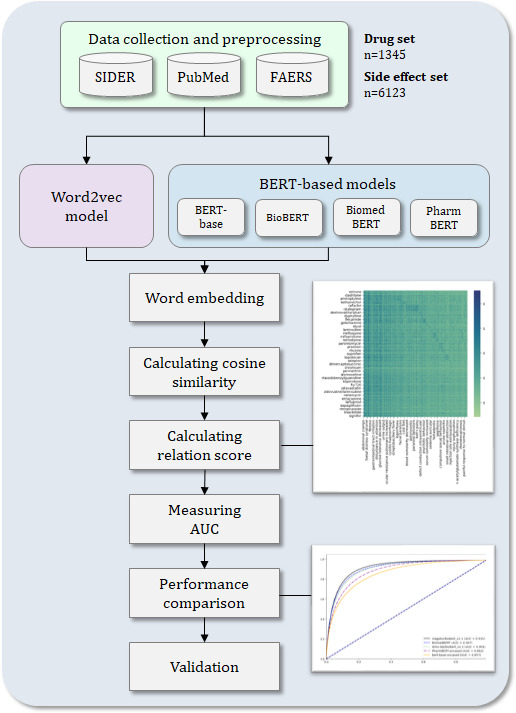
System overview: the predictive performance of drug-side effect relationships was evaluated using area under the curve. AUC: area under the curve; BERT: Bidirectional Encoder Representations from Transformers; FAERS: FDA (Food and Drug Administration) Adverse Event Reporting System; SIDER: side effect resource.

Figure 1 presents an overview of this study and illustrates the overall research flow from data collection to validation.

First, we collected and refined the data for this study from the Side Effect Resource (SIDER), PubMed, and the FDA Adverse Event Reporting System (FAERS). From the abstract sentences collected from PubMed, we selectively extracted only sentences containing drugs and side effects mentioned in SIDER. These extracted sentences were used to train a Word2Vec model, from which embedding vectors for drugs and side effects were derived. Based on the drug–side effect relationships in SIDER, BERT-based models were used to derive the embedding vectors of drugs and side effects.

Based on the derived embedding vectors, the cosine similarity between the drug and side effect pairs was calculated. Using the cosine similarity of drug and side effect pairs and existing known drug–side effect relationships, a relation score was calculated for all drug–side effect combinations. Drug–side effect combinations with high relation scores were predicted to have a higher likelihood of being actually related [12]. To evaluate the accuracy of these predictions, we calculated area under the curve (AUC) values and compared the results of the Word2Vec model pipeline with those of the BERT-based model pipeline. Statistical significance between predicted results and FAERS was assessed using the Fisher exact test.

### Data Collection and Preprocessing

SIDER is a database that provides information on marketed drugs and their side effects [28]. The drug names recorded in SIDER followed those approved by the Food and Drug Administration (FDA), and side effect names used the Medical Dictionary for Regulatory Activities (MedDRA) terminology [29]. To minimize data leakage that could occur from the same drug being listed under different names, we collected and integrated synonyms for each drug using PubChem compound identifiers provided by SIDER. We also ensured terminology standardization through MedDRA-based side effect names.

**Figure 2.**
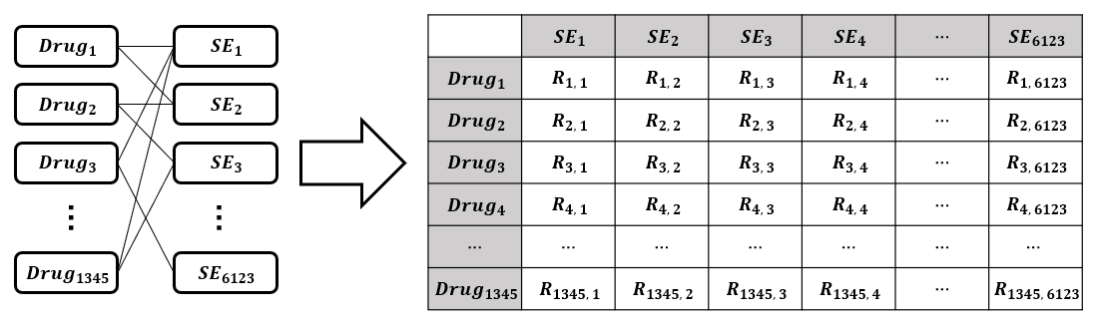
Drug-SE adjacency matrix. This is a method to derive an adjacency matrix using drug–side effect relationships in the side effect resource. The relation R has the value of 1 if the drug–side effect relationship exists and 0 if it does not. SE: side effect.

Using version 4.1 of SIDER, we collected 158,096 unique pairs of drug–side effect relationships after removing duplicates. To use these 158,096 pairs as input values in the BERT-based models and for relation score calculations, we derived an adjacency matrix with drugs as rows and side effects as columns (Figure 2). In addition, 1345 drug names and 6123 side effect terms that appeared in the collected drug–side effect relationships were extracted and used as dictionaries for drugs and side effects.

We collected biomedical literature from PubMed, a biological literature database [30]. A total of 42,515,246 paper abstracts updated on December 8, 2022, were collected, and for training the Word2Vec model, only sentences in which the drugs and side effects mentioned in SIDER were mentioned at least once were extracted [12]. There were 14,289,160 sentences in which a drug was mentioned at least once and 32,107,327 sentences in which a side effect was mentioned at least once.

### Calculating Cosine Similarity

For the 1345 drugs and 6123 side effects recorded in the adjacency matrix, we performed word embedding using BERT-based models and calculated the cosine similarity for all drug and side effect vector pairs. In this case, the cosine similarity is calculated using equation (1).


$$CosineSimilarity=\frac{A\cdot B}{\left\|A\right\|\left\|B\right\|}=\frac{\sum_{i=1}^{n}A_{i}\times B_{i}}{\sqrt{\sum_{i=1}^{n}{\left(A_{i}\right)}^{2}}\times \sqrt{\sum_{i=1}^{n}{\left(B_{i}\right)}^{2}}}\left(1\right)$$

This process yielded 1,809,025 drug vector pairwise similarities and 37,491,129 side effect vector pairwise similarities.

### Calculating Relation Score

**Figure 3.**
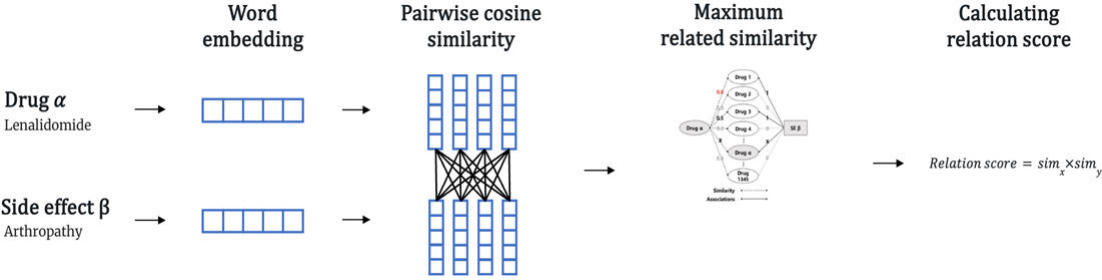
Illustration of the computation of the relation score between a specific *Drug*_*α*_ and a specific side effect *SE*_*β*_.


Figure 3 shows the process of calculating the relation score. For all drug–side effect pairs embedded as vectors, the cosine similarity values obtained in the previous step were used to calculate the drug–side effect’s relation score. The process of calculating the relation score between a specific *Drug*_*α*_ and a specific side effect *SE*_*β*_ was done using equations (2) to (6).

$$si\;m_{x}=max\{∼(D_{a},D_{i})\vee \;D_{i}\;\in \;Related_{Drugs}\}\;(2)$$

$$Related_{Drugs}=\{D_{i}\vee Adjacency(SE_{\beta },D_{i})=1\;for\;i=1,2,...,1345\}\;(3)$$

$$si\;m_{y}=max\{∼(S\;E_{\beta },S\;E_{i})\;\vee S\;E{\;}_{i}\;\;\in Related_{SE}\}\;(4)$$

$$Related_{SE}=\{S\;E_{i}\vee Adjacency\;(D_{a},S\;E_{i})=1\;for\;i=1,2,...,6123\}\;(5)$$

$$Relation\;score=si\;m_{x}\times si\;m_{y}\;(6)$$

Similarity *sim_x_* takes the maximum of the similarity values of *Drug*_*α*_ with Drug *D*_*i*_ in *Related*_*Drugs*_, the set of drugs known to be associated with side effect *SE*_*β*_, using equation (2). The set *Related*_*Drugs*_ is obtained using equation (3), and by referring to the values in the adjacency matrix consisting of 1345 drugs and 6123 side effects, we construct the set of drugs associated with that side effect by including in the set *Related*_*Drugs*_ those drugs that have side effect SE, and a value of 1 in the adjacency matrix, out of a total of 1345 drugs. In other words, the highest similarity value to drugs known to be associated with side effect SE is called similarity *sim*_*x*_.

**Figure 4.**
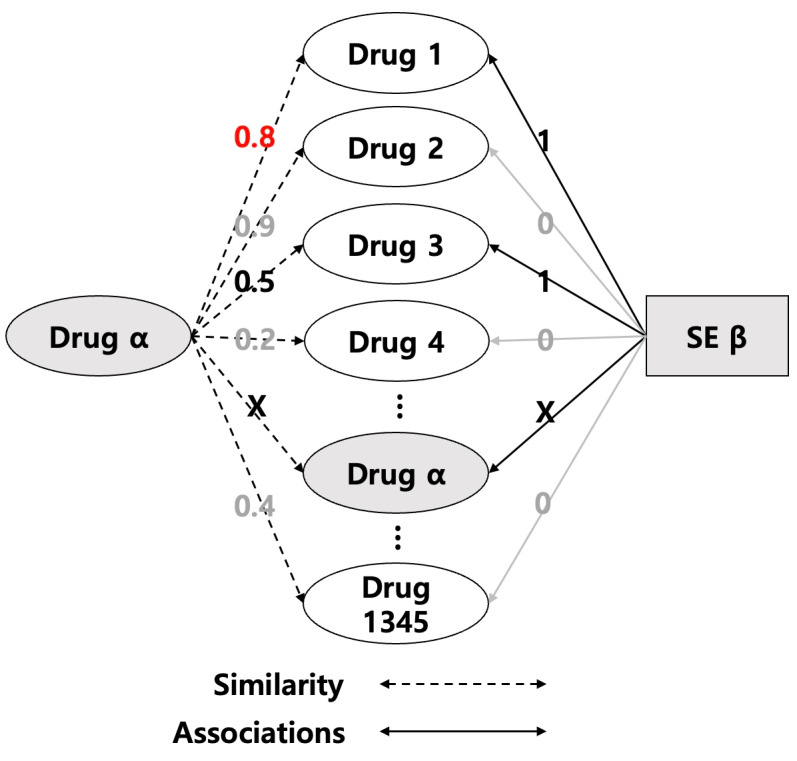
Process of calculating similarity *sim*_*x*_. SE: side effect.

Figure 4 shows the process of obtaining similarity *sim*_*x*_ using equation (2) and quation (3). Based on the values in the adjacency matrix, the computational process was to maximize the similarity of only those drugs that were related to the side effect SE out of the total 1345 drugs. If *Drug*_*α*_ is in the *Related*_*Drugs*_ set, exclude *Drug*_*α*_ from the similarity calculation.

Similarity *sim*_*y*_ uses equation (4) to take the maximum of the similarity values of side effects *SE*_*β*_ and *SE*_*i*_ in *Related*_*SE*_, a set of side effects known to be related to the *Drug*_*α*_. The set *Related*_*SE*_ is obtained using equation (5), and by referring to the values of the adjacency matrix mentioned above, we construct the set of side effects associated with the *Drug*_*α*_ out of the total 6123 side effects by including in the set *Related*_*SE*_ the drugs that have a value of 1 in the adjacency matrix with the *Drug*_*α*_. In other words, the highest similarity value to the side effects that are known to be related to the drug is called the similarity *sim*_*y*_.

**Figure 5.**
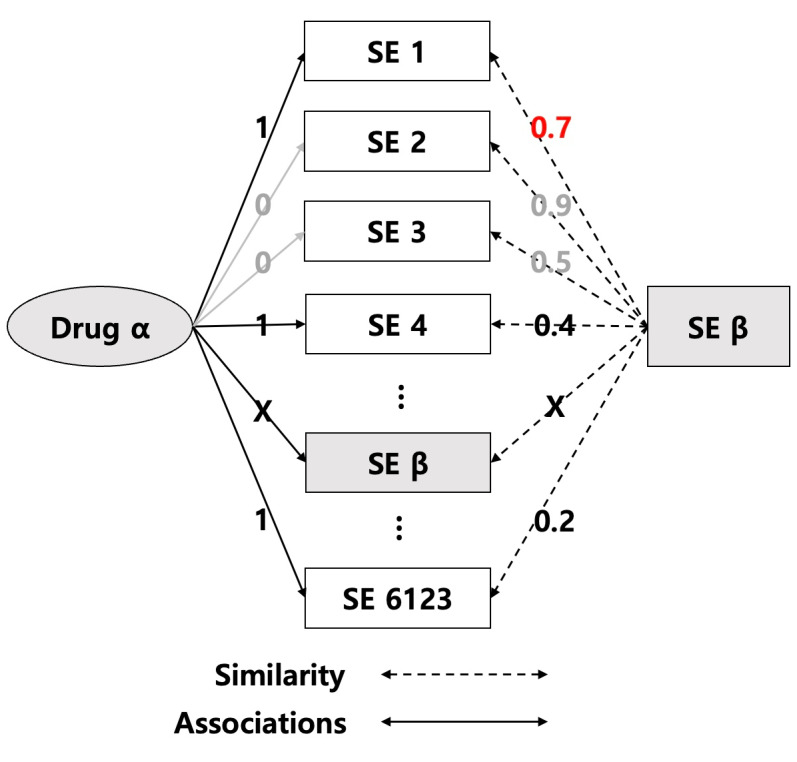
Process of calculating similarity *sim*_*y*_. SE: side effect.

Figure 5 shows the process of obtaining similarity *sim*_*y*_ using equation (4) and equation (5). Based on the values in the adjacency matrix, the computational process is to extract the similarity of only the side effects that are related to the *Drug*_*α*_, out of the total 6123 side effects, and take the maximum value. If an *SE*_*β*_ belongs to the *Related*_*SE*_ set, exclude the *SE*_*β*_ from the similarity calculation.

Finally, the relation score between Drug and SE was obtained by multiplying *sim*_*x*_ and *sim*_*y*_ as shown in equation (6).

**Figure 6.**
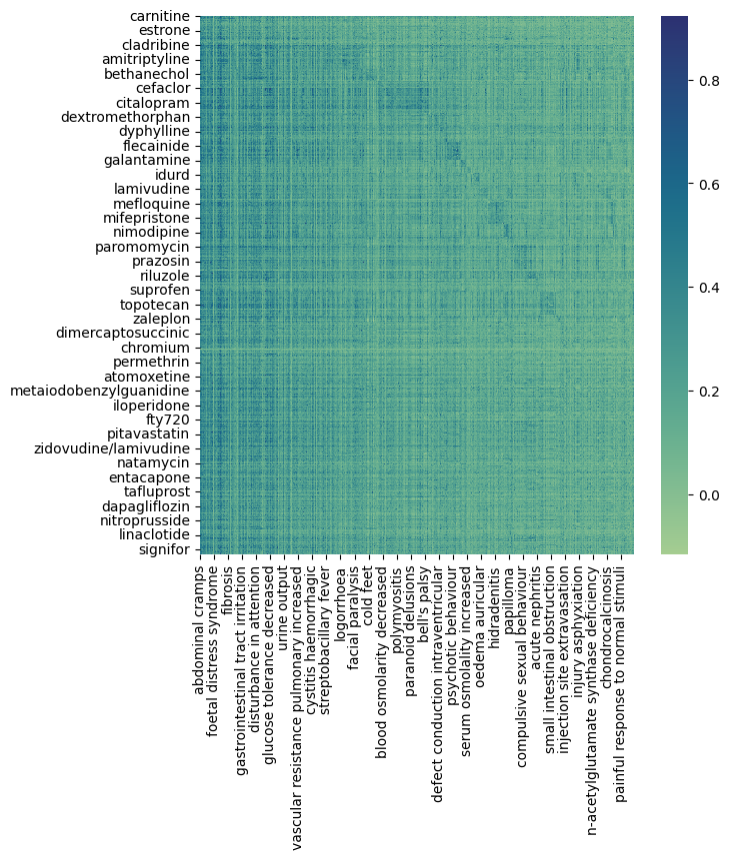
Heatmap of calculated relation score for 8,235,435 drug–side effect pairs.

We applied the above calculation method to 1345 drugs and 6123 side effects to calculate the relation scores for all drug–side effect pairs, resulting in a total of 8,235,435 drug–side effect pairs. Figure 6 illustrates the heatmap of the calculated relation scores.

### Measuring AUC

In this study, AUC values were measured using 158,096 known drug–side effect relationships provided by SIDER to evaluate the accuracy of predicting relationships based on scores calculated for a total of 8,235,435 drug–side effect pairs. Of the 8,235,435 calculated drug–side effect pairs, we assigned a class value of true to pairs that belonged to known drug–side effect relationships in SIDER and false to pairs that did not. All drug–side effect pairs were sorted by score, and a single receiver operating characteristic curve was calculated. The generated receiver operating characteristic curves and AUC values were used to establish the optimal threshold for predicting whether a drug–side effect pair had a true relationship. If the drug–side effect relation score exceeds this diagnostic threshold, it is predicted that there is a relationship between the drug and the side effect [12].

### Ethical Considerations

This study uses the publicly accessible and anonymized SIDER and FAERS databases, which contain no personally identifiable information and do not involve human participant experimentation. Therefore, institutional review board approval is not required for this study.

## Results

### Performance Comparison

**Figure 7.**
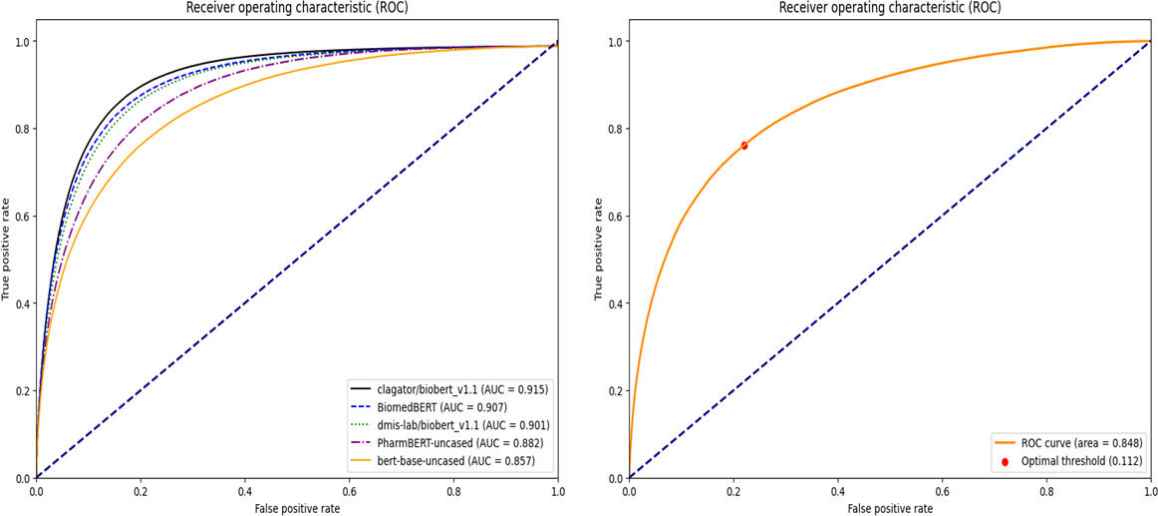
Receiver operating characteristic curves for Bidirectional Encoder Representations from Transformers (BERT)–based models (left) and Word2Vec model (right). AUC: area under the curve.

The AUCs of the BERT-based models and the Word2Vec model using the proposed method are compared in Table 1 and Figure 7. The optimal threshold for prediction was set as the point at which the sum of the sensitivity and specificity was maximized. The models used in this study included clagator/biobert_v1.1 [31], BiomedBERT [20], dmis-lab/biobert_v1.1 [19], PharmBERT-uncased [23], bert-base-uncased [17], and Word2Vec [11]. dmis-lab/biobert-v1.1 is the original BioBERT model pretrained on biomedical text, while clagator/biobert_v1.1 is a model based on it, which has been additionally fine-tuned on natural language inference and semantic textual similarity tasks to enhance its ability to recognize semantic relationships.

**Table 1.** Performance comparison of Bidirectional Encoder Representations from Transformers (BERT)–based models and Word2Vec model.

Model	AUC^a^	Optimal threshold	Sensitivity	Specificity
clagator/biobert_v1.1	0.915^b^	0.289	0.870	0.830
BiomedBERT^c^	0.907	0.925	0.857	0.821
dmis-lab/biobert_v1.1	0.901	0.780	0.851	0.814
PharmBERT-uncased	0.882	0.460	0.817	0.796
bert-base-uncased^d^	0.857	0.617	0.769	0.793
Word2Vec	0.848^e^	0.112	0.762	0.780

a AUC: area under the curve.

b Highest value.

c The old model was named PubMedBERT.

d Vanilla BERT model.

e Lowest value.

The clagator/biobert _v1.1 model achieved the highest AUC value of 0.915 at an optimal threshold of 0.289. In contrast, the bert-base-uncased model, a vanilla BERT model pretrained on general corpora, showed an AUC of 0.857 at an optimal threshold of 0.617. In other words, BERT pretrained on the biomedical corpus outperformed vanilla BERT. In addition, the Word2Vec model recorded an AUC of 0.848, which was lower than those of the BERT-based models and was the lowest among all models used in this study.

**Figure 8.**
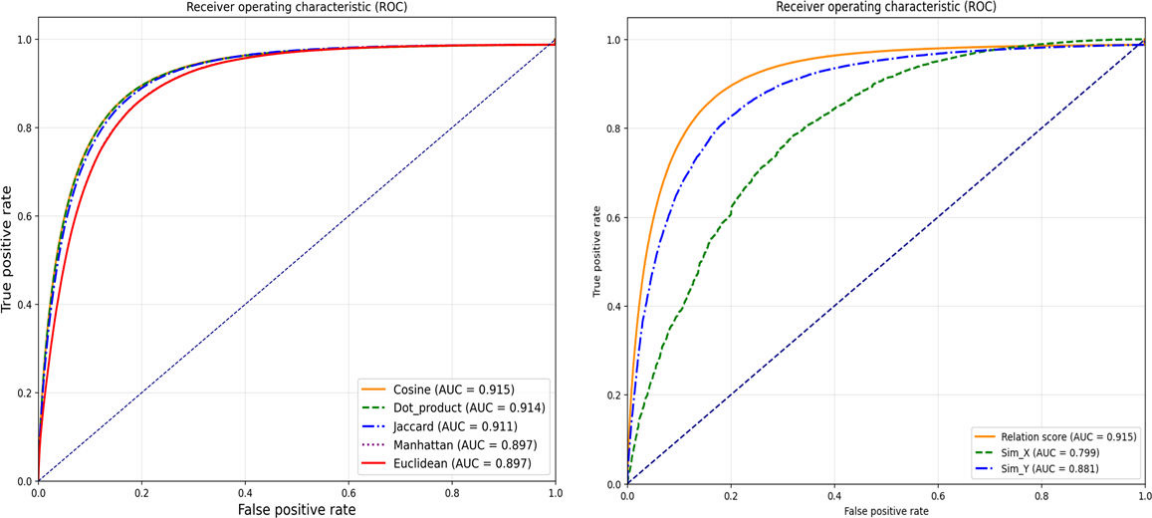
Receiver operating characteristic curves for comparing vector similarity metrics (left) and evaluating the efficacy of our dual-similarity approach (right). AUC: area under the curve.

Figure 8 (left) shows the performance comparison results to evaluate the effectiveness of the cosine similarity-based extraction approach. The comparison was conducted using the clagator/biobert_v1.1 model, which achieved the highest performance. Other vector similarity-based extraction methods used for comparison included Euclidean distance, Manhattan distance, Jaccard similarity, and dot product. The results demonstrate that the cosine similarity-based relation extraction method used in this study exhibited the highest performance with an AUC of 0.915, outperforming all other methods.

Figure 8 (right) evaluates the effectiveness of our dual-similarity approach against single-similarity methods. Using the same clagator/biobert_v1.1 model, we compared 3 extraction strategies: using only drug similarity information (Sim_X), using only side effect similarity information (Sim_Y), and our dual-similarity approach (relation score). Our dual-similarity approach significantly outperformed single-similarity methods with an AUC of 0.915 compared with 0.799 and 0.881, demonstrating the effectiveness of leveraging information from both similarity perspectives.

### Validation

To validate the similarity between drug–side effect relationships predicted by our model based on SIDER data and relationships derived from FAERS data, we extracted drug–side effect relationships from FAERS, a database not used in our Methods. FAERS is a database containing adverse event information for drugs submitted to the FDA [32]. In this study, we used FAERS data from October 2012 to June 2023. By leveraging the list of 1345 drugs and 6123 side effects registered in SIDER to extract relationships from FAERS data, we obtained a total of 901,361 known relationships.

For validation, we used the results from clagator/biobert_v1.1 [31], which performed best in our study. We constructed a contingency table using the predicted results based on relation scores from 8,235,435 drug–side effect pairs, along with 901,361 known drug–side effect relationships and unknown relationships extracted from FAERS. However, due to the data imbalance where unknown relationships outnumbered known relationships, we randomly sampled unknown relationships to match the number of known relationships. Based on this selected list of drug–side effect pairs, we conducted the Fisher exact test between FAERS data and our study’s predictions, repeating this process 2000 times and calculating the average of all results. The results showed a *P* value of *P*<.001, confirming that the drug–side effect relationships predicted in our study were statistically significant.

Furthermore, the odds ratio, calculated as the ratio of the odds of an event occurring to the odds of it not occurring, was 4.822. This means that the odds of our model predicting a relationship for known relationships in FAERS were 4.822 times higher than the odds of predicting a relationship for unknown relationships. In other words, relationships reported in FAERS were significantly more likely to be predicted as related by our model, demonstrating that our model’s predictions are reliable when compared with external data.

To validate the utility of our model’s predictions, we conducted case studies on drug–side effect relationships not present in the SIDER database that were ranked within the top 1000 according to relation scores calculated by our model. For these candidates, we performed literature searches using Google Scholar and verified whether these relationships had been mentioned in case reports or research [33]. In addition, following the input from an author specializing in pharmacoepidemiology, we excluded steroid-class drugs that are used despite their side effects from a clinical utility perspective, thereby enhancing the reliability of our ability to detect meaningful signals in actual clinical environments. Table 2 illustrates the cases of lenalidomide-arthropathy, rosuvastatin-sleep disturbance, gadolinium-acute pulmonary edema, and cefazolin-hepatic failure. Upon conducting a literature review of the drug–side effect relationships presented in Table 2, we confirmed that these associations were reported in research findings published after 2016 [34-37].

**Table 2.** Case studies of model predictions.

Drug *α*	Side effect *β*	Similarity *x*	Similarity *y*	Relation score	Model prediction
Lenalidomide	Arthropathy	0.953	0.837	0.799	True
Rosuvastatin	Sleep disturbance	0.952	0.825	0.786	True
Gadolinium	Acute pulmonary edema	0.891	0.866	0.771	True
Cefazolin	Hepatic failure	0.861	0.857	0.738	True

## Discussion

### Principal Findings

In this study, we propose a method to extract information about drug–side effect relationships inherent in the pre–trained parameters of language models and predict relation scores, indicating the possibility of unknown drug–side effect relationships. This is accomplished using known drug–side effect relationship data and embedding vectors from language models trained on biomedical corpora.

Our study confirmed that BERT-based models demonstrated superior performance in predicting drug–side effect relationships. We evaluated the performance of BERT-based models using the relation score methodology proposed by Seungsoo et al [12], and the clagator/biobert_v1.1 model [31] achieved the highest performance with an AUC of 0.915 at an optimal threshold of 0.289. This suggests that BERT-based models perform better in predicting drug–side effect relationships compared to the 0.85 AUC achieved by the Word2Vec model in a previous study. Therefore, our findings support the notion that context-aware BERT-based models outperform context-independent Word2Vec models in terms of embedding performance [38].

In addition, our study demonstrates that BERT models pretrained on biomedical corpora outperform vanilla BERT models pretrained on general corpora. Vanilla BERT models, trained on general corpora, have limitations in fully reflecting the specificity of the biomedical field [20]. In contrast, BERT-based models pretrained on large biomedical corpora, such as PubMed and PMC, more richly reflect drug mechanisms of action and biological relationships observed in clinical settings [19-21]. The results of this study demonstrate that BERT models specialized for biomedical applications can provide more accurate drug–side effect relationship predictions based on a deeper understanding of the domain. This aligns with previous studies that emphasize the importance of domain-specific models in BERT model applications [19,23,39]. While this study evaluated the effectiveness of the proposed extraction method using BERT-based models, considering the rapid advancements in the field of NLP, models with different architectures or more recent models have the potential to understand the complexity of relationships more effectively and provide further performance improvements.

We performed external validation using FAERS data and found a high statistical significance (*P*<.001) between 8,235,435 predicted drug–side effect relationships and 901,361 actual data extracted from FAERS. In addition, to verify the real-world applicability of the model’s predicted results, we conducted case studies on drug–side effect relationships that were not confirmed in the SIDER database. We found that these drug–side effect relationships have been reported in recent research findings published after 2016. This suggests that our methodology using the BERT-based model proposed in this study is applicable to the prediction of ADRs in practice. Considering this, we expect that our proposed methodology will allow for earlier detection of potential ADRs, increasing the likelihood of success in the drug development process and reducing the time and cost of ADR studies.

In the field of biomedical NLP, standardized terminology systems and synonym processing are important [15,40]. In this study, we minimized the risk of data leakage by integrating drug synonyms using PubChem compound identifiers. We used MedDRA-based side effect names to ensure terminology standardization and reduce the likelihood of the model merely reidentifying variations of known relationships. However, we have not explicitly integrated the hierarchical information from MedDRA, and in future research, we plan to enhance our methodology by actively using MedDRA hierarchical information to integrate similar terms, further mitigating the reidentification issue.

In this study, we recognize that non–dose-dependent adverse reactions present particular challenges for prediction models, and drug similarity may not necessarily be the main component in the development of such ADRs. While our dual-similarity approach partially addresses this by incorporating adverse event similarity patterns, future research would benefit from integrating NLP-based approaches with the chemical structure and mechanism of action of drugs. This multimodal approach would leverage literature-derived contextual relationships, molecular properties, and biological pathway insights to more accurately classify drug–side effect relationships and would be particularly valuable for addressing current methodological limitations, including novel compound prediction and idiosyncratic reaction detection.

### Limitations

One of the primary limitations of this study is the lack of up-to-date data in the drug–side effect database used. The SIDER database was last updated in 2015, meaning that despite using BERT-based models trained on the latest biomedical corpus, our prediction process may not fully reflect current drug–side effect relationships. Consequently, incorporating more recent drug–side effect data would likely improve the performance of our prediction model significantly.

Another significant limitation relates to the nature of case reports themselves. Such reports typically rely on a single clinically reported case, thereby making it difficult to establish clear causal relationships between drugs and side effects. In addition, their small sample sizes often limit their generalizability. These considerations become particularly relevant when extending our work to clinical applications, where patient care involves complex interactions of multiple factors, including diverse reporting patterns and polypharmacy. Although our model provides a systematic method for prioritizing potential associations for further investigation, all predictions should therefore be interpreted with appropriate caution and validated through additional pharmacovigilance methods before clinical application. For this reason, future research should utilize systematic clinical data or large-scale cohort studies to enhance the reliability of predictive models.

Furthermore, while our approach shows promise for identifying potential drug–side effect relationships through vector space similarities, we recognize 2 additional important limitations. First, the current methodology provides generalized, population-level predictions and does not account for idiosyncratic reactions dependent on individual patient factors. As such, future work should explore integrating patient-specific data to enable more personalized adverse event predictions. Second, for entirely novel drug candidates absent from existing literature, the embedding vectors generated would be based primarily on semantic inference rather than established knowledge, potentially limiting prediction reliability. This underscores the importance of complementary approaches, particularly for new chemical entities.

### Conclusions

This study presents a novel approach for extracting drug–side effect relationship information embedded within pretrained language model parameters and leveraging this information to predict unknown adverse reactions. Our methodology, using context-aware BERT-based language models, demonstrates that BERT models pretrained on biomedical corpora outperform vanilla BERT and Word2Vec. These results highlight how the contextual embedding capabilities of BERT architectures, coupled with domain-specific adaptation, enhance predictive performance in drug–side effect relationship tasks. Furthermore, external validation using FAERS data and a literature review of selected cases confirmed the practical applicability of the proposed methodology.

## Abbreviations

ADR: adverse drug reaction
AUC: area under the curve
BERT: Bidirectional Encoder Representations from Transformers
FAERS: FDA Adverse Event Reporting System
FDA: Food and Drug Administration
MedDRA: Medical Dictionary for Regulatory Activities
NLP: natural language processing
SIDER: Side Effect Resource
